# Psychometric evaluation of the computerized battery for neuropsychological evaluation of children (BENCI) among school aged children in the context of HIV in an urban Kenyan setting

**DOI:** 10.1186/s12888-023-04880-z

**Published:** 2023-05-29

**Authors:** Maina Rachel, He Jia, Abubakar Amina, Miguel Perez-Garcia, Manasi Kumar, Jelte M. Wicherts

**Affiliations:** 1 Department of Methodology and Statistics, Tilburg University, Tilburg, Netherlands; 2 Brain and Mind Institute, Aga Khan University, Nairobi, 10834-00400 Kenya; 3 Institute for Human Development, Aga Khan University, Nairobi, Kenya; 4 Mind, Brain and Behavior Research Center (CIMCYC), University of Granada, Granada, Spain

**Keywords:** Cognitive tests, Validity, Reliability, The BENCI, Kenya, School aged children, HIV

## Abstract

**Introduction:**

Culturally validated neurocognitive measures for children in Low- and Middle-Income Countries are important in the timely and correct identification of neurocognitive impairments. Such measures can inform development of interventions for children exposed to additional vulnerabilities like HIV infection. The Battery for Neuropsychological Evaluation of Children (BENCI) is an openly available, computerized neuropsychological battery specifically developed to evaluate neurocognitive impairment. This study adapted the BENCI and evaluated its reliability and validity in Kenya.

**Methodology:**

The BENCI was adapted using translation and back-translation from Spanish to English. The psychometric properties were evaluated in a case–control study of 328 children (aged 6 – 14 years) living with HIV and 260 children not living with HIV in Kenya. We assessed reliability, factor structure, and measurement invariance with respect to HIV. Additionally, we examined convergent validity of the BENCI using tests from the Kilifi Toolkit.

**Results:**

Internal consistencies (0.49 < α < 0.97) and test–retest reliabilities (-.34 to .81) were sufficient-to-good for most of the subtests. Convergent validity was supported by significant correlations between the BENCI’s Verbal memory and Kilifi’s Verbal List Learning (*r* = .41), the BENCI’s Visual memory and Kilifi’s Verbal List Learning (*r* = .32) and the BENCI’s Planning total time test and Kilifi’s Tower Test (*r* = -.21) and the BENCI’s Abstract Reasoning test and Kilifi’s Raven’s Progressive Matrix (*r* = .21). The BENCI subtests highlighted meaningful differences between children living with HIV and those not living with HIV. After some minor adaptions, a confirmatory four-factor model consisting of flexibility, fluency, reasoning and working memory fitted well (χ^2^ = 135.57, *DF* = 51, *N* = 604, *p* < .001, RMSEA = .052, CFI = .944, TLI = .914) and was partially scalar invariant between HIV positive and negative groups.

**Conclusion:**

The English version of the BENCI formally translated for use in Kenya can be further adapted and integrated in clinical and research settings as a valid and reliable cognitive test battery.

**Supplementary Information:**

The online version contains supplementary material available at 10.1186/s12888-023-04880-z.

## Introduction

Human Immunodeficiency Virus (HIV) is a neurotropic virus that can infect the nerve cells [1]. Widespread access to antiretroviral drugs (ARVs) has reduced the severity of HIV related brain diseases [2]. However, even when children are on ARVs and virologically suppressed, they may continue to manifest neurocognitive impairments [3–5]. The monitoring of neurocognitive performance among children with HIV should be included in a comprehensive HIV management plan [6, 7]. However, in sub-Saharan Africa (SSA) the lack of adequately standardized neurocognitive tools that are easy to implement [2] at a relatively low cost inhibits the implementation of recommended neurocognitive monitoring among HIV-positive children. To address this gap in health care, it is important to identify and validate neurocognitive measures that can be easily implemented in health care settings within the African setting. Given how limited the resources are in many of these settings, neurocognitive tools for use in SSA need to be open-access and relatively easy to administer so that they can be implemented by para-professionals or professionals with limited training. These tools should also be engaging to the children.

In recent years, there has been a proliferation of computerized neurocognitive tools which are relatively easy to implement, yet many of these tools have largely been developed and tested in high-income countries [6]. They include the NIH toolbox, Conner’s Continuous Performance Test, Attentional Network Task (ANT), CNS Vital Signs and Pediatric Immediate Post Concussion Assessment and Cognitive Testing (Pediatric ImPACT) [8–13]. Due to potential measurement biases that may arise from adopting test from one context to another, it is crucial that these new promising tests are thoroughly evaluated in the SSA context [2, 14–16]. Here, we study the psychometric properties and potential utility of the computerized Battery for Neuropsychological Evaluation of Children (The BENCI) which covers several neuropsychological domains [17] and was originally developed in Spanish for Ecuadorian children. The BENCI measures the seven cognitive domains with the following subtests: Simple Reaction Time, Visuo-motor, Continuous Performance, Verbal Memory, Visual Memory, Verbal Comprehension Images, Verbal Comprehension Figures, Phonetic Fluency, Working Memory, Abstract Reasoning, Semantic Fluency, Go/NO-GO, Spatial Stroop, Alternate Visuo-motor, and Planning-Attraction Park tests [18]*.* See Table 1 for their specific domains and administration. The fact that the BENCI is openly available and computerized makes it relatively easy to access and administer. It is also enjoyable for children [19], hence curtailing for loss of interest and distraction, which may result in low completion rates, missing responses, and erroneous responses.

**Table 1:** BENCI and Kilifi toolkit tests

BENCI (90 min)			KILIFI TOOLKIT (120 MINUTES)		
**Domain**	**Sub-test**	**Outcome Measures**	**Domain**	**Sub-test**	**Outcome Measures**
Processing Speed	Simple Reaction Time Test (a plus sign of the screen prompts the child to press a key on the keyboard fast)	Mean RT & Median RT	-	-	
Visuo-motor Coordination	Visuo-motor test (involves connection of elements/number in a given sequence)	TT & Total Errors			
Sustained Attention	Continuous Performance Test (respondent presses any key every time the required stimulus appears)	Hits/CA, EO, EC, Mean RT & Median RT	Visual Sustained and Selective Attention Auditory Sustained and Selective Attention	People Search (A stimulus sheet comprising complete and incomplete stick figures is presented. The subject is required to cross out only complete figures, as quickly as possible) Digit span as we could not find the tape. The child is instructed to repeat a series of numbers (with increasing numbers of digits) forward. Each correct response is worth one point; with a maximum of 14 points for each sub-score series	TT, RT, Errors of Omission (EO) and Errors of Commission (EC) TT and Highest Score
Memory	Verbal memory test (child listens to some words then repeats the ones remembered) Verbal memory delayed recall test (the series of words said are repeated after 20 min) Verbal Memory Essay of Recognition test (words are read out loud and respondents identifies those that were in the previous list) Visual memory (series of images are presented after which respondents verbalizes those remembered) Visual Memory delayed Essay (the images remembered are said out loud after 20 min) Visual Memory Essay of Recognition (respondent identifies if images presented were in previous list)	Hits/CA, P & I Hits/CA, P & I Hits/CA & Errors Hits/CA, EC & EO Hits/CA, EC & EO Hits/CA, EC & EO	Memory	Working Memory: Verbal List Learning – VLL (Two lists of 15 items are read out to the child as a shopping list. The first is presented five times and the second only once) Subtests within include: - Verbal Memory Test Free Recall Trial Test Short Delay Free Recall Trial Short Delay Cued Recall Trial Long Delay Free Recall Trial Long Delay Cued Recall Trial Long Delay Recognition Trial	Intrusions (I), Perseverations (P), CA and TT
Language	Verbal Comprehension Images Test (respondent matches images to given conditions) Verbal Comprehension Figures (respondent matches geographic shapes to given conditions) Phonetic Fluency (a letter is presented and respondents verbalizes all words that start with the letter given.)	Hits/CA & Errors Hits/CA & Errors Hits/CA, I & P	-	-	
Executive Functioning	Working Memory (a list of color and numbers are said and respondent repeats the numbers then the colors) Abstract Reasoning (respondent completes a logical series by selecting the right element) Semantic Fluency (a category is given and respondents says the elements known in that category) Inhibition: Go/NO-GO (respondents identifies distinguishing factor between two elements and later identify the distinguishing element) Flexibility: Spatial Stroop (respondent matches arrow directions to arrow labels) (Two components of spatial stroop—attention shifting task measures flexibility while proper spatial stroop task measures inhibition) Flexibility: Alternate Visuo-motor (is flexibility measure that involves two distinct series in which the respondent should connect alternatively) Planning: Attraction Park (respondent chooses a number of attractions according to money in hand with each attraction chosen expiring after a given period)	Hits/CA Hits/CA Hits/CA, I & P EC, Hits/CA & Mean RT Median RT, EC, EO TT & Total Errors Planning Time, TT, Rule 1, Rule 2, fairground amusements & different fairground amusements/CA	Executive Functioning	Self-Ordered Pointing Test—SOPT (Selection of pictures displayed in varying positions on separate sheets in sets of 6, 8, 10, and 12. As each page is turned the subject is required to identify all members of the set, but to point to each item of the set only once. Touching a picture more than once is considered an error) Raven progressive matrices: Reasoning: Colored Progressive Matrices – CPM (Three sets with 12 matrices made of abstract patterns. The subject is asked to complete the matrix by placing one of a choice of four patterns in the empty space) Attention and attention shift: Contingency Naming Test – CNT (The child is taught a series of rules to name nine drawings displayed in a single series. Each drawing consists of a large outer colored shape and a smaller inner colored shape. Each drawing is named according to the shape or color of one of its two shapes. The rules taught for selecting the name of the item become more complex over four trials) Planning: Tower Test/ Tower of London (Three colored wooden balls are moved between three pegs to match a goal position. Time and number of moves required are recorded**)**	Time Taken(TT), Reaction Time(RT) and Correct Answers (CA)

*RT* Reaction Time, *TT* Total Time, *CA* Correct Answers, *EO* Errors of Omission, *EC* Errors of Commission, *I* Intrusions, *P* Perseverations

Since the BENCI is a promising tool with its psychometric properties already documented in Morocco among 7, 9, and 11 year old children in schools, its adaptation and implementation in Kenya among children living with HIV and children who are HIV negative can expand our school-age children toolbox and provide clinics with rigorously validated measures [20]. Data from Moroccan children supported a factorial structure of executive functioning with inhibition, flexibility, fluency, reasoning, and verbal memory in the Arabic version of the BENCI [20]. In deciding the executive function tests to include in the factorial model, the previous study acknowledged the lack of a theoretical model that could explain the battery’s structure. Hence, we opted to use Diamond model’s [21] of executive functioning to create our model. We included verbal tests as indicators of executive function because tests of verbal memory [22, 23] have been associated with executive function outcomes with up to 55–60% shared variance [22]. However, factorial structure and measurement invariance with respect to HIV status has yet to be evaluated in a similar LMIC region. Measurement invariance evaluates whether the subtests are loaded similarly onto the latent factors and whether groups based on, e.g., educational attainment, health status, ethnicity and age can be meaningfully compared [24]. Since the language of instruction in the Kenyan schools is English [25] we choose to adapt an English version of the BENCI. Moreover, computerized assessment is rare in Kenya, and this study with the computerized BENCI is an important first step to assess the feasibility of reliably evaluating neurocognitive functions using computerized measures in the Sub-Saharan context. To conduct a comprehensive evaluation of the BENCI, we carried out the following:

1. Adapted the BENCI in a culturally appropriate adaptation format and user-centered testing
2. Evaluated its internal consistency and test–retest reliability
3. Examined the associations between the results of the BENCI (a computerized test) and those of a paper-and pencil standardized test
4. Evaluated differences in performance and measurement properties among children who are living with HIV versus those who are not living with it.

## Methodology

### Participants and settings

A total of 604 (311 females, 291 males and two with missing gender information) children from Nairobi participated in the study. Nairobi is the capital city of Kenya with a 87.1% literacy level and the language of instruction in the schools is English [25]. We recruited two samples from different study sites. One group of children was sampled from a children HIV outpatient programme. The programme, implemented in seven resource poor settings in Nairobi, included children living with HIV of different ethnic backgrounds who receive home-based care. The sample of children not living with HIV was drawn from three primary schools in Nairobi. The schools were chosen on the basis of their similarity to most schools in Kenya with regards to the mode of education at that time which was the 8.4.4 system with the examining body under the Ministry of Education being the Kenya National Examination Council [26]. These children come from diverse socio-economic settings with most of them from middle-class families. We chose this to rule out the impact of sharp socioeconomic status differences. The study sample size computation was based on data from an earlier study in Africa that found the means on the KABC – 2 to differ between HIV-infected (*N* = 93) and uninfected (*N* = 106) [27] by ${\mu }_{1}=184.7 \left(sd=63.72\right)\mathrm{ and}{ \mu }_{2}=200.6 \left(sd=68.72\right)$, respectively, yielding a Cohen’s d of 16.1/66.3 = 0.24. Together with an alpha level of 5% and a power of 80%, these resulted in a total sample size of 544 respondents, thus the target sample size was 272 children living without HIV and 272 children living with HIV, respectively. We slightly oversampled to address any potential loss of data due to missingness.

### Measures

*The BENCI*: The existing BENCI test was first developed in Ecuador and offers norms for children aged 6 – 17 years in Ecuador, 7, 9 and 11 years in Morocco and 6—8 years in Palestine [18, 19]. The test can be administered within 75 min with one 10-min break in between the 14 neuropsychological tests. On average, however, the administration takes around 90 min. The test can be administered by skilled psychologists with additional training specific to BENCI.

*Paper and Pencil Measures:* To test convergent validity of the BENCI, we used paper and pencil tests that are internationally accepted and standardized and have previously been adapted and validated in a rural Kenyan community [28]. This so-called Kilifi Toolkit covers executive functioning, memory, and attention and can be administered within 120 min. The neurocognitive tests have good psychometric properties with *split- half reliability between 0.70 and 0.84 while internal consistency is* ≥ *0.70* among 7 – 11 year old children in Kenya [28]. Table 1 lists tests in Kilifi toolkit and the BENCI. As part of our study, we also measured age, gender, height, and weight.

#### The BENCI Adaptation process

The adaptation process was guided by the translation and adaptation guidelines of the International Test Commission [29]. We obtained authorization to adapt the original BENCI test and the original test developers including MPG who also had an advisory role in test adaptation. Since the original BENCI was in Spanish, the translation was the first stage of adaptation where one bilingual researcher translated it from Spanish to English and another native English speaker checked the English translation for linguistic and semantic consistency. Clinical psychologists in Kenya, in discussions with other professionals in Spain, evaluated the tools’ structure and appropriateness against the tool’s original markers in terms of sentence structure and familiarity of images in the Kenyan context. This work was complemented by a pilot study involving 5 females and 3 males with a median age of 13 years to check the appropriateness of the items, pictures, and instructions. The pilot study involved administering all the sub tests within the BENCI and later interviewing each child individually on how they experienced the tests.

*In terms of the BENCI administration*, some children expressed that the sustained attention test was too lengthy which lowered their enthusiasm for doing the rest of the tests. This was discussed with the study team and changes were made to place the sustained attention test right before the 10 min’ break. Children tended to touch the screen with their fingers playfully even when not responding and this resulted in unintended responses especially in the Visual Memory and Verbal Memory with Delayed Trial test. Hence, BENCI administrators were instructed to caution the children against moving their hands on the screen if they did not have any intention to respond.

*In language,* some English words in the instructions of some BENCI subtests were unclear to some young children. An example is the word ‘figures’ which was changed to ‘shape’ as Kenyan children are more familiar with the latter than the former. Some instructions were not clear enough, hence recommendations were made to ensure that children understood what to do when a certain stimulus appeared, especially in the verbal comprehension subtest. In the Continuous performance test, instructions on pressing screen right after letter X appeared after letter A were not clear. We therefore agreed that we would draw a letter A followed by letter X to help in indicating when the screen should be pressed. Several instructions were changed to simpler English. Young children had a better understanding of the test requirements when additional information was given in Kiswahili – the national language of Kenya.

**Fig. 1:**
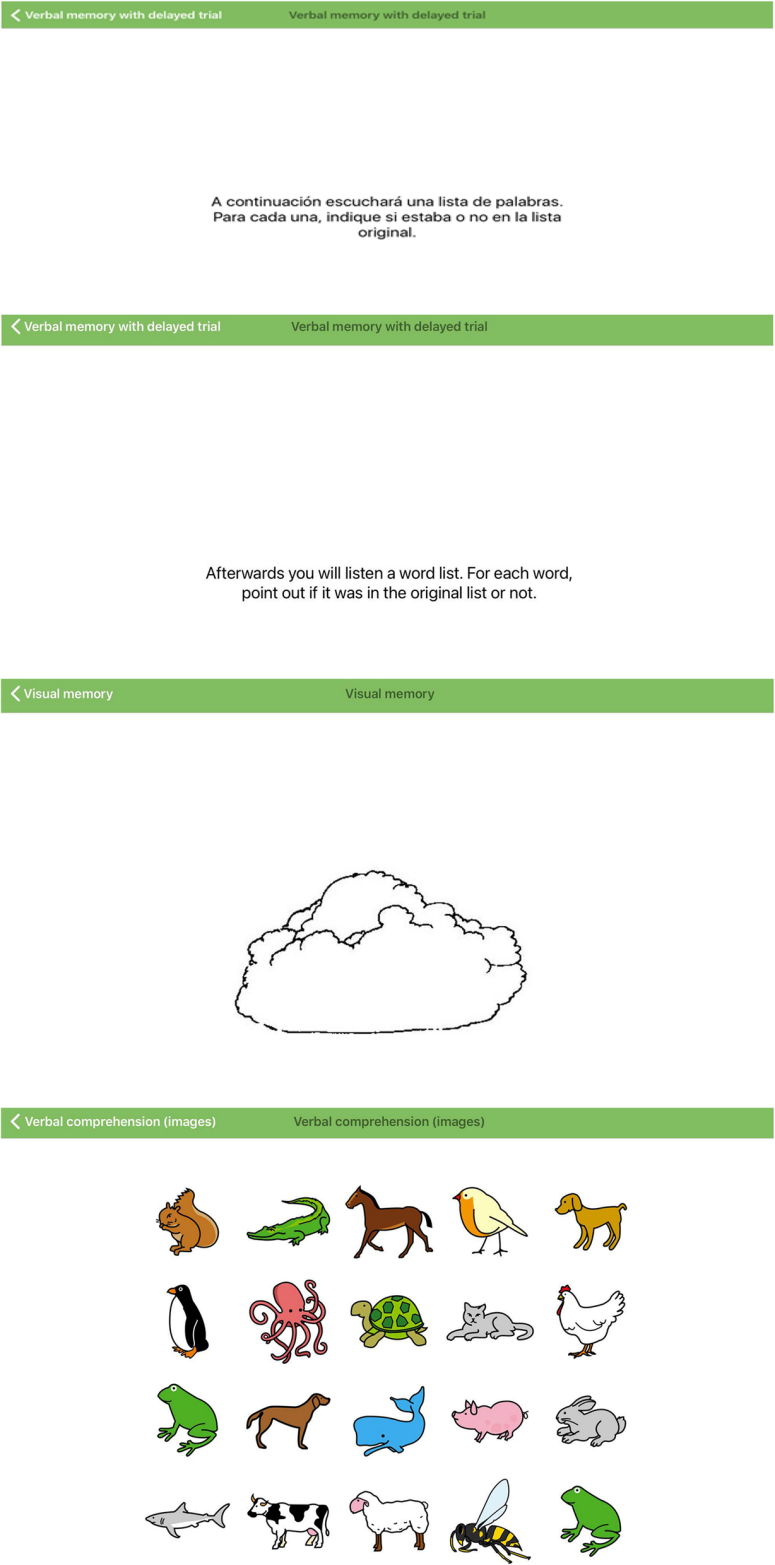
Translation and cultural adaptations made in BENCI

*Cultural adaptations* were also made to images in the verbal comprehension test, as young children did not recognize some animals like the difference between a squirrel and a rabbit, while some animals had some striking resemblance to animals familiar to the Kenyan children. Images within the visual memory subtest, which could not be recognized by children, were also changed, or scoring changed to include the interpretation that was familiar to the children. For example, some children could not differentiate between cloud and bush as the images were similar so both answers were integrated as the correct answers in the scoring guide. See Fig. 1 for the pictorial presentations on the changes made in the BENCI.

### Procedure

In the clinics, a database of children aged 6 – 14 years old was generated and the children were informed to come to the clinic on a certain day of the week when the programme arranged for some fun activities to take place. Most of the time the assessment day fell on a weekend and on the same day as the children were scheduled for their clinical appointments. On the scheduled day, the children and their parents were randomly identified and individually informed about the study with voluntary participation of the children being requested. We included children aged 6–14 years^1^ that are HIV-positive and not having any comorbid conditions as reported in their medical reports. We did not include children with comorbid and/or severe medical conditions associated with being HIV-positive as indicated in their medical reports, as well as children who did not meet the age criteria. In the school setting, the children were randomly selected from their classrooms, which ranged from Grades 1 to 5. In this population, we included children aged between 6 – 14 years old and not having any medical condition as reported by the school and the students themselves. Children who did not meet these conditions were excluded from the study. The institutions provided a room where the neurocognitive assessments could be carried out. Relevant subtests in Kilifi toolkit (see Table 1) were administered with paper and pencil by a trained interviewer [28]. For test–retest reliability, 38 HIV negative children (21 females) were re-assessed 2 months after the initial assessment.

### Analyses

Data from the BENCI was automatically captured in the tablet as programmed in the original Spanish version and exported to an Excel sheet. The Kilifi Toolkit data were input into Excel sheets and codes/matching identifications were realigned to ensure correct matching with similar cases in the file with BENCI data. We double-checked the age, gender, and clinic/school groupings to ensure the correct ID matching. Analyses were run in SPSS version 20 and AMOS version 22. We used Alpha = 0.05 as the nominal significance level.

Data were cleaned by first having a visual inspection of a scatter plot and statistical evaluation of each of the subtest scores for outliers. Data with influential outliers were then evaluated through a three-step process to identify if certain scores should be deleted. First, we checked the residuals of the regression of age on the subtests where cases with high standardized residual value, low effect size, and low p-value were noted. Second, we evaluated cases with *z* scores beyond z =|2| for possible deletion. Third, we conducted a case-by-case check to evaluate whether a certain score would be expected given other subtest scores from these participants. For instance, we discarded scores on the Verbal Memory Immediate hits and Continuous Performance hits subtest that had z-scores below -3 and whose z- scores were not expected for the age groups we were looking at. Through this process, we decided whether certain scores should remain as they are or identify them as missing. We then carried out a missing data pattern analysis where Little's MCAR test statistic was significant (χ^2^ = 2455.2, DF = 1725, *p* < 0.001), highlighting that scores were not missing completely at random. However, a check on whether the missingness was significantly related to age, HIV status, and date of data collection, uncovered no significant relationship with the missingness pattern in subtest scores. We could, therefore, not identify what the missingness was related to.

Internal consistency in terms of Cronbach’s Alpha (KR-20 for dichotomous items) was determined for all seven tests for which item-level data were available. We opted for Cronbach’s Alpha because it is widely used in testing the internal consistency of the items within a test that reflects the degree to which items covary positively. The test–retest reliability was analyzed using ICC and Pearson’s correlations. We then checked whether the performance within the BENCI subtests aligned with developmental models’ expectation of growth in cognitive performance as children grew in age. Convergent validity was analyzed using Pearson correlation where scores of the BENCI subtests were correlated with the raw scores of corresponding subtests in Kilifi Toolkit. We hypothesized that tests measuring the same cognitive domain would correlate positively. We compared differences between HIV-positive and HIV-negative groups with t-tests and considered possible floor and ceiling effects by checking histograms and outliers by calculating skewness for each subtest.

We run a confirmatory factor analysis in AMOS to assess the construct validity the BENCI using a model of Executive Function proposed by Diamond, in which executive function comprises reasoning, inhibition, flexibility, fluency, and working memory cognitive functions [30]. The model fit was evaluated with the Chi-square tests, Root Mean Square Error of Approximation (RMSEA), Comparative Fit Index (CFI), and the Tucker-Lewis Index (TLI). A model is considered a good fit if the value of RMSEA is below 0.06, and CFI and TLI above 0.90 respectively.

## Results

The two test batteries were administered among 274 children living with HIV and 330 children without HIV with a mean age of 9.48 (SD = 1.31), of which roughly half were male. Table 2 summarizes the demographics of participants in the two groups. The second assessment of the BENCI among 38 Children not living with HIV consists of 21 females, with a mean age of 9.18 (*SD* = 1.21).

**Table 2:** Socio demographic information

Variables		HIV Negative N (%)	HIV Positive N (%)
Gender	Male	163 (49.40)	148 (54.00)
	Female	166 (50.30)	125 (45.60)
	Missing	1 (0.30)	1 (0.40)
Age in months (Mean ± SD)		117.2 ± 16.24	119.40 ± 14.63
Age in Years (Mean ± SD)		9.41 ± 1.37	9.56 ± 1.24
Nutrition	Weight in kg (Mean ± SD)	34.98 ± 7.12	32.27 ± 5.85
	Height in cm (Mean ± SD)	136.34 ± 8.00	133.02 ± 8.11

### Scale attenuation effects

Using correlational and descriptive statistics including histograms, we evaluated attenuation patterns in the BENCI tests. Eight of the BENCI subtests exhibited ceiling and floor effects that tend to suppress correlations and reliabilities. Specifically, on Verbal Comprehension Figures, 30% (*N* = 181) of the sample scored the highest possible score of 8 hits, while on Verbal Comprehension Images hits, 51% (*N* = 308) of the sample scored the highest possible score of 8 hits. Other subtests with ceiling effects included *Continuous Performance hits, Go No Go hits, Working Memory hits,* and *Spatial Stroop.* Both *Verbal Memory Recognition* 13.4% (*N* = 16) and *Visual Memory Recognition* 16.7% (*N* = 20) showed some ceiling effects meaning that the number of participants having the highest scores was almost equal to those with average scores. At the same time, floor effects were evident on the *Planning Time of First Option* and *Spatial Stroop errors scores*. *Semantic Fluency hits 13.4% (N* = *16), Phonetic Fluency hits 16% (N* = *19), Verbal Memory Delayed hits* 16.8% (*N* = 20), and *Planning time total 33.9% (N* = *38)* showed some floor effects. This meant that the number of participants having the lowest scores was almost as equal to those with average scores. The floor and ceiling effects highlighted that these subtests psychometric functioning could be improved by adding easier and more difficult items, respectively, in any future revisions of the BENCI. The remaining BENCI subtests showed no such attenuation effects.

### Internal consistency of the BENCI

We computed the Cronbach’s Alphas (KR-20 s) for seven of the subtests with dichotomous item scores. The internal consistency of the BENCI subtests varied from poor to excellent reliability. As shown in Table 3, the Language Comprehension tests, Verbal Comprehension Images, and Figures, had the fewest items (*N* = 8) and Cronbach Alpha 0.49 < α < 0.68 which was the lowest among the other BENCI subtests. Low Cronbach Alphas tend to suppress correlations, but most of the BENCI subtests had high Alphas. The Abstract reasoning, Planning, Go No Go, Spatial Stroop, and Processing Speed tests correlated well with themselves (0.75 < α < 0.97 or alpha range from 0.75 to 0.97) hence showing that there was little random measurement error.

**Table 3:** BENCI items internal consistency

BENCI Subtests	No. of Items	Skewness	Overall Cronbach's Alpha	HIV Negative Cronbach's Alpha	HIV Positive Cronbach's Alpha
Verbal comprehension images Time	8	3.113	.689	.519	.682
Verbal comprehension images Hits	8	-1.302	0.592	0.386	0.602
Verbal comprehension figures Time	8	1.926	0.613	0.56	0.609
Verbal comprehension figures Hits	8	-.737	0.496	0.349	0.571
Abstract reasoning Hits	25	.019	0.832	0.813	0.781
Abstract reasoning Time	25	.851	0.890	0.904	0.875
Go No Go Total Hits	101	-.745	0.870	0.824	0.895
Go No Go Total Time	101	1.136	0.879	0.872	0.864
Planning Total time	12	.895	0.753	0.760	0.744
Spatial Stroop Hits	90	-.933	0.973	0.966	0.975
Spatial Stroop Time	90	-1.701	0.950	0.924	0.959
Processing speed Reaction Time	50	1.598	0.832	0.832	0.822

Possibly due to the ceiling effects being less severe because of lower mean scores, we found Verbal Comprehension Figures and Images tests to show higher internal consistencies among children living with HIV (0.57 < α < 0.68) than among children not living with HIV (0.35 < α < 0.56), whose scores were more affected by the ceiling effect. In the Abstract reasoning, Planning, Go No Go, Spatial Stroop, and Processing Speed sub-tests the items had acceptable and excellent (0.76 < α < 0.97, or alpha range from 0.76 to 0.97) internal consistency showing that the tests are reliable for both children living with HIV and those not living with HIV, as shown in Table 4. The Alphas in the latter tests were higher in the lower-scoring sample of children living with HIV than in children not living with HIV due to less severe attenuation effects in the former group.

**Table 4:** Reliability test–retest of the BENCI battery

Test (N = 38)	First Visit Mean (sd)	Second Visit Mean (sd)	ICC	CI 95%	Pearson correlation
Visuomotor Coordination (TT)	73,772.32 (34,587.11)	54,539.74 (27,326.12)	.66**	.35—.82	.51**
Alternate Visuomotor Coordination (TT)	75,473.65 (32,581.90)	50,385.71 (23,478.43)	.74**	.49—.87	.62**
Sustained Attention CPT (CA)	49.06 (12.06)	51.70 (6.26)	.13	-.74—.57	.08
Sustained Attention CPT (RT)	626.96 (191.41)	618.57 (196.13)	.81**	.62—.90	.68**
Immediate Verbal Memory (CA)	5.19 (2.60)	6.26 (3.38)	.58*	.12—.77	.39*
Delayed Verbal Memory (CA)	5.30 (3.01)	5.58 (3.00)	.71**	.44—.85	.55**
Verbal Recognition Memory (CA)	18.89 (3.49)	20.00 (3.08)	.41	-.15—.70	.26
Immediate Visual Memory (CA)	5.76 (2.60)	6.30 (3.29)	.75**	.51—.87	.61**
Delayed Visual Memory (CA)	5.35 (3.22)	6.47 (3.29)	.55*	.13—.77	.38*
Visual Recognition Memory (CA)	44.30 (6.08)	45.47 (4.11)	.52*	.07—.75	.38*
Comprehension of Images (CA)	7.53 (0.97)	7.78 (0.42)	.49*	-.01—.74	.45*
Working Memory (CA)	11.58 (5.69)	13.76 (4.92)	.71**	.43—.85	.55**
Reasoning (CA)	13.89 (4.11)	15.74 (4.39)	-.34	-1.63—.32	-.15
Semantic Fluency (CA)	8.00 (3.01)	6.84 (3.58)	.64*	.30—.81	.48*
Phonetic Fluency (CA)	4.89 (2.48)	5.68 (2.83)	.48*	-.00—.73	.32
Go/No-Go (CA)	0.87 (0.14)	0.84 (0.16)	.43*	-11—.71	.27
Go/No-Go (RT)	0.64 (0.08)	0.66 (0.11)	.14	-.71—.56	.08
Selective Attention (RT)	575.21 (148.28)	573.16 (153.80)	.66**	.32—.83	.49*
Planning FO (RT)	5047.03 (5865.45)	2600.13 (3168.40)	.43*	-.11—.71	.32*

### Tests retest reliability of the BENCI

Table 4 presents the Intraclass Correlation (ICC) of the test and retest scores of the BENCI and the Pearson correlations between the repeated measurements among the 38 children not living with HIV. The Intraclass correlation for specific tests ranged from -0.34 to 0.81. The coefficients were rather high in Sustained Attention RT, Immediate Visual Memory, and Alternate Visuo-motor Coordination (*ICC range from 0.74 to 0.81, r* = *0.68—0.62).* Moderate correlations were found in Immediate Verbal Memory, Delayed Visual Memory, and Visual Recognition Memory *(ICC range from 0.52 to 0.58, r* = *0.39—0.38).* Test retest reliability was poor for Go/No-Go (RT), Sustained Attention CA, and Reasoning *(ICC range from 0.14 to -0.34, r* = *0.08—-0.15).*

The test–retest reliability results showed that most of the tests were consistent on the two occasions (2 months in between t1 and t2). With clear significant gains in performance as expected by increasing test familiarity and maturation for fifteen out of nineteen subtests, except for Sustained Attention CPT, Verbal Recognition Memory (CA), Reasoning (CA), and Go/No-Go (RT) that showed no clear improvements in mean performance.

### Convergent validity

Table 5 presents the correlations between corresponding BENCI and Kilifi toolkit tests. The attention, memory, inhibition/planning, reasoning, and flexibility tests in the BENCI and Kilifi were expected to correlate. However, some of these tests did not correlate as expected due attenuation effects, while others correlated as expected despite the attenuation effects.

**Table 5:** BENCI – Kilifi toolkit convergent validity

**BENCI Tests**	**Kilifi Toolkit Tests**										
		**People Search test**	**Digit Span test**	**Contingency Naming test**	**Self-Ordered Pointing Test**	**Verbal List Learning (VLL) test – Total CA**	**Nonverbal Selective Reminding Memory Test (NVSRT)**	**Tower Test**	**Ravens Progressive Matrices test**	**VLL Immediate Memory Span**	**VLL Level of Learning**
	*Domains*	*Visual Sustained and Selective Attention*	*Auditory Sustained and Selective Attention*	*EF: Flexibility—Attention and attention shift*	*EF: Working Memory*	*Memory*	*Non-Verbal Memory*	*EF: Inhibition—Planning*	*EF: Reasoning*	*Memory*	*Memory*
**Sustained Attention CPT (CA)**	*Sustained Attention*	-0.103	0.053	-0.157	0.093	.303^b^	0.089	0.056	.288 ^b^	0.056	.266 ^b^
**Sustained Attention CPT (RT)**	*Sustained Attention*	0.123	-0.024	0.130	-0.043	-0.110	0.123	0.066	-0.151	0.066	-0.062
**Working Memory (CA)**	*EF: Working Memory*	.194 ^a^	-0.124	-0.047	0.004	.276 ^b^	0.085	-0.143	0.049	-0.143	.297 ^b^
**Verbal memory (CA)**	*Memory*	-0.006	0.005	-0.030	0.014	.414 ^b^	-0.181	-0.165	.346 ^b^	-0.165	.372 ^b^
**Verbal Memory Delayed (CA)**	*Memory*	-0.038	0.043	-0.089	0.116	0.193	-0.102	-0.171	0.162	-0.171	.212 ^a^
**Verbal Memory Recognition (CA)**	*Memory*	-0.010	-0.025	-0.001	0.068	-0.076	0.012	-.186^a^	-0.073	-.186 ^a^	-0.085
**Planning Total Time**	*EF: Inhibition—Planning*	-0.083	-0.176	.279^b^	-0.011	-0.030	-.405 ^b^	-.209^a^	-0.010	-.209^a^	0.004
**Planning Time FO**	*EF: Inhibition—Planning*	-0.169	-0.128	.219 ^a^	-0.070	-0.060	-.310 ^b^	-0.113	0.047	-0.113	-0.028
**Reasoning (CA)**	*EF: Reasoning*	-.367 ^b^	0.119	0.042	0.000	.424 ^b^	-.279 ^b^	-0.087	.206^a^	-0.087	.380 ^b^
**Visual Memory Immediate (CA)**	*Memory*	0.041	0.012	0.010	-0.081	.322 ^b^	-0.112	0.060	0.119	0.060	.234 ^a^
**Visual Memory Delayed (CA)**	*Memory*	0.038	-0.033	-0.007	0.064	.252 ^a^	-0.088	-0.179	.261 ^a^	-0.179	0.220
**Visual Memory Recognition (CA)**	*Memory*	-0.109	0.215	-0.057	0.092	0.129	-0.022	0.008	0.078	0.008	0.047
**Spatial Stroop Flexibility**	*EF: Flexibility*	-0.142	0.086	0.027	0.084	.414 ^b^	-.202^a^	-0.037	.327 ^b^	-0.037	.361 ^b^

*TT* Total Time, *RT* Reaction Time, *CA* Correct Answers

^b^ Correlation is significant at the 0.01 level (2-tailed)

^a^ Correlation is significant at the 0.05 level (2-tailed)

In domains of reasoning, several inhibition, and a few memory-related tests in the BENCI were positively correlated with tests in Kilifi toolkit, supporting convergent validity across these domains. The BENCI’s Working Memory test was expected to correlate with Kilifi’s Self-Ordered Pointing Test (SOPT) because they both measure working memory. However, the BENCI Working Memory test did not have a significant correlation with Kilifi’s working memory test, Self-Ordered Pointing Test (SOPT). This could be because the BENCI Working Memory test showed ceiling effects and might have been too easy for most test takers.

Kilifi’s Verbal List Learning Test and Nonverbal Selective Reminding Memory test were expected to correlate with the BENCI’s Verbal Memory and Visual Memory tests because they all measure memory. However, none of the BENCI’s memory tests had a significant correlation with Kilifi’s Nonverbal Selective Reminding Memory Test (NVSRT). Moreover, the BENCI’s Verbal Memory Recognition and Visual Memory Recognition tests had no significant correlation to any of Kilifi’s memory tests. This outcome could be because the BENCI’s Verbal Memory Recognition and Visual Memory Recognition tests had some ceiling effects while Kilifi’s NVSRT had floor effects. However, the BENCI’s Verbal Memory Immediate hits had a significant correlation with Kilifi’s Verbal List Learning’s (VLL) Immediate Memory Span (*r* = 0.37), Level of Learning (*r* = 0.40) and Total correct answers (*r* = 0.41). In addition, the BENCI’s Verbal Memory Delayed Trial was also significantly correlated with Kilifi’s Verbal List Learning’s Immediate Memory Span (*r* = 0.21). Moreover, the BENCI’s Visual Memory Immediate hits had a significant correlation with Kilifi’s Verbal List Learning’s (VLL) Immediate Memory Span (*r* = 0.23), Level of Learning (*r* = 0.34) and Total correct answers (*r* = 0.32). In addition, BENCI’s Visual Memory Delayed Trial was also significantly correlated with Kilifi’s Verbal List Learning’s (VLL) Level of Learning (*r* = 0.23) and Total correct answers (*r* = 0.25). The significance was found despite the BENCI’s Verbal Memory Delayed showing some floor effects. The rest of the memory tests in the BENCI and Kilifi had no ceiling or floor effects. The correlation between Kilifi’s Verbal List Learning’s (VLL) Level of Learning and Total correct answers and the BENCI’s Reasoning test was not expected. As expected, the BENCI Abstract Reasoning Test significantly correlated with Kilifi’s Raven’s Progressive Matrix (RPM) (*r* = 0.21). Both reasoning tests had no attenuation effects.

Kilifi’s People Search test and FNRT test were expected to correlate with BENCI’s Continuous Performance test and Spatial Stroop Attention test because they all measure attention. Among the attention tests, the BENCI sustained attention test, Continuous Performance hits and reaction time test, did not have a significant correlation with Kilifi’s visual sustained and selective attention—People Search test (*r* = -0.10; *r* = 0.12), as well as auditory sustained and selective attention test—Forward Digit Span total score (*r* = -0.14; *r* = 0.07). People Search test had floor effects while Continuous Performance hits had ceiling effects. Moreover, the BENCI tests that contain an attention component, Reasoning (*r* = -0.37) and Working Memory (*r* = 0.19) were also significantly correlated to Kilifi’s People Search. Kilifi’s People Search and its correlation with the BENCI’s Reasoning and Working Memory tests was unexpected as these BENCI tests are not primarily meant to measure attention.

BENCI’s Spatial Stroop was expected to correlate with Kilifi’s Contingency Naming test (CNT) because they both measure flexibility. However, the Spatial Stroop test, had no significant correlation with the Contingency Naming test (CNT) (*r* = 0.03). The Spatial Stroop test showed ceiling effects while CNT had no attenuation effects.

Kilifi’s Tower Test was expected to correlate with the BENCI’s planning test because they both measure inhibition. This is indeed the case, as the BENCI Planning Total Time test had a significant association with Kilifi’s Tower test (*r* = -0.21). However, BENCI’s Planning Time of First Option test had no significant association with Kilifi’s Tower test (*r* = -0.11). This results should be interpreted cautiously because the BENCI’s Planning Total Time test had some floor effects while the Planning Time of First Option had floor effects indicating that items were relatively difficult for our test takers.

Overall, in the reasoning domain, much convergence between the BENCI and Kilifi Toolkit was supported, whereas in the memory and inhibition domains there was only partial convergence. Subtests in the flexibility, attention, and working memory domains showed little convergent validity with the Kilifi mostly because of attenuation effects.

### The BENCI functionality in age and HIV groups

As can be seen in Table 6, children not living with HIV outperformed those living with HIV on all BENCI tests. However, the mean group difference was significant in all subtests except Continuous Performance Test hits and reaction time, Go No Go hits, Verbal Memory Recognition hits, and Planning total time.

**Table 6:** Mean group differences in BENCI subtests responses

		**N**	**Mean**	**Std. Deviation**	**Std. Error Mean**	**Mean Difference**	**Significance (2-tailed)**
Verbal Comprehension Figures Hits	HIV negative	317	7.030	.990	.056	.498	.000
	HIV positive	258	6.530	1.171	.073		
Verbal Comprehension Images Hits	HIV negative	318	7.540	.743	.042	.760	.000
	HIV positive	259	6.780	1.220	.076		
Continuous performance Hits	HIV negative	322	47.329	12.756	.712	1.928	.097
	HIV positive	264	45.401	14.891	.916		
Continuous performance RT Median	HIV negative	318	585.997	149.796	8.400	20.093	.061
	HIV positive	260	565.904	107.263	6.652		
Go No Go Hits	HIV negative	315	42.333	8.007	.451	.287	.709
	HIV positive	258	42.047	9.968	.621		
Go No Go Mean RT	HIV negative	315	.825	.007	.000	-.002	.023
	HIV positive	252	.827	.009	.001		
Processing Speed Median Reaction Time	HIV negative	313	584.931	146.541	8.283	20.6036	.048
	HIV positive	267	564.328	102.179	6.253		
Phonetic Fluency Hits	HIV negative	319	5.100	2.729	.153	1.658	.000
	HIV positive	271	3.440	2.546	.155		
Semantic Fluency Hits	HIV negative	321	7.310	3.295	.184	1.909	.000
	HIV positive	271	5.410	3.440	.209		
Working Memory Hits	HIV negative	319	10.510	6.060	.339	1.955	.000
	HIV positive	270	8.560	6.314	.384		
Verbal Memory Hits	HIV negative	264	6.240	2.249	.138	.747	.000
	HIV positive	206	5.500	2.040	.142		
Verbal Memory Hits Delayed	HIV negative	317	4.670	3.192	.179	1.125	.000
	HIV positive	270	3.540	2.706	.165		
Verbal Memory Hits Recognition	HIV negative	317	18.310	3.533	.198	.517	.089
	HIV positive	270	17.790	3.810	.232		
Planning Total Time	HIV negative	303	18,178.770	12,791.017	734.825	148.873	.891
	HIV positive	259	18,029.900	12,776.036	793.864		
Planning Time of First Option	HIV negative	304	3404.050	3027.831	173.658	-567.889	.036
	HIV positive	259	3971.940	3316.305	206.065		
Abstract Reasoning Hits	HIV negative	319	14.870	4.835	.271	4.261	.000
	HIV positive	269	10.610	4.775	.291		
Visual Motor Total time	HIV negative	320	70,080.740	28,797.814	1609.847	-18,559.339	.000
	HIV positive	254	88,640.080	35,451.218	2224.407		
Alternative Visual-Motor Total Time	HIV negative	315	74,940.830	36,611.539	2062.827	-22,239.897	.000
	HIV positive	255	97,180.730	45,435.071	2845.254		
Visual Memory Immediate Hits	HIV negative	278	6.140	2.295	.138	1.277	.000
	HIV positive	207	4.860	2.030	.141		
Visual Memory Delayed Hits	HIV negative	273	6.150	2.459	.149	1.289	.000
	HIV positive	201	4.860	1.990	.140		
Visual Memory Recognition Hits	HIV negative	314	44.580	5.122	.289	1.527	.003
	HIV positive	264	43.050	6.975	.429		
Spatial Stroop Hits	HIV negative	328	66.500	21.956	1.212	7.270	.000
	HIV positive	270	59.230	23.769	1.447		
Spatial Stroop Omission Errors	HIV negative	328	9.050	10.469	.578	-5.962	.000
	HIV positive	270	15.010	16.667	1.014		
Spatial Stroop Commission Errors	HIV negative	328	11.710	15.242	.842	-2.719	.032
	HIV positive	270	14.430	15.571	.948		
Spatial Stroop Mean Time	HIV negative	328	979.504	223.457	12.338	-68.133	.001
	HIV positive	270	1047.638	249.922	15.210		

We checked whether the performance within the BENCI subtests aligned with developmental models’ expectation of growth in cognitive performance as children aged, and report Pearson correlations between age in years and the BENCI subtest performance for the children living with HIV- and those not living with HIV separately in Table 7. We hypothesized that children not living with HIV would significantly outperform those living with HIV. Among the children living with HIV, there were significant associations in the expected direction between age and Verbal Comprehension Images hits, Verbal Memory hits, Verbal Memory Recognition hits, planning total time, Planning Time of First Option, Abstract Reasoning hits, Visual Memory Immediate hits, Visual Memory Recognition hits and Spatial Stroop omission errors. Among children not living with HIV, there was a significant association between age and Continuous Performance reaction time, Processing Speed reaction time, Verbal Memory hits, Abstract Reasoning hits, and Visual Memory Delayed hits. The lack of significant correlations between some cognitive indicators and age could be because of attenuation effects, but might also relate to sampling issues (e.g., older participants appearing in the sample because of delayed development and the repeating of grades in school).

**Table 7:** Age correlations in BENCI subtests responses

	**HIV Positive**			**HIV Negative**		
	**Pearson Correlation**	**Sig. (2-tailed)**	**N**	**Pearson Correlation**	**Sig. (2-tailed)**	**N**
Age in years			252			292
Verbal comprehension Images Hits	.188^b^	.004	239	.038	.520	283
Continuous Performance Hits	-.050	.435	244	-.004	.945	288
Continuous Performance RT Median	-.054	.409	240	-.168^b^	.004	285
Go No Go Total Hits	.074	.253	239	-.023	.698	281
Go No Go Mean RT	-.113	.085	233	.048	.422	281
Processing Speed Median Reaction Time	-.123	.053	247	-.156^b^	.009	279
Phonetic Fluency Hits	-.063	.321	250	.034	.571	284
Semantic Fluency Hits	.025	.689	250	.006	.921	286
Working Memory Hits	-.046	.468	249	-.100	.094	284
Verbal Memory Hits	.156^a^	.032	190	.255^b^	.000	234
Verbal Memory Hits Delayed	.048	.450	249	-.006	.921	282
Verbal Memory Hits recognition	.151^a^	.017	249	.034	.564	282
Planning Total Time	.128^a^	.049	238	-.015	.803	267
Planning Time of First Option	.237^b^	.000	240	.006	.922	269
Abstract Reasoning Hits	.156^a^	.014	248	.210^b^	.000	284
Visual Motor Total Time	-.126	.055	233	-.083	.161	285
Alternative Visual Motor Total Time	.105	.108	237	-.006	.914	281
Visual Memory Immediate Hits	.160^a^	.027	191	.082	.196	248
Visual Memory Delayed Hits	.096	.195	185	.230^b^	.000	243
Visual Memory Recognition Hits	.150^a^	.019	245	.037	.541	279
Spatial Stroop Hits	.106	.095	249	.034	.561	292
Spatial Stroop Omission Errors	-.152^a^	.017	249	-.082	.163	292
Spatial Stroop Commission Errors	-.013	.838	249	.003	.964	292
Spatial Stroop Mean Time	-.017	.788	249	-.072	.218	292

^b^. Correlation is significant at the 0.01 level (2-tailed)

^a^. Correlation is significant at the 0.05 level (2-tailed)

### Confirmatory factor analyses

We tested the construct validity of Executive Functioning as proposed by Diamond for normal development [30]. According to his model, the subtests that measure inhibition, flexibility, reasoning, memory, and fluency together constitute executive functioning [30]. These are tests that evaluate the ability to make decisions, exercise self-control, pay attention, be creative, solve problems, and plan towards having good health and success in life. These are considered core functions in the brain hence the name executive functions. We fitted a confirmatory factor analysis model previously fitted successfully in the Arabic version of the BENCI [20] and sought to adjust the model slightly to improve fit if necessary.

**Fig. 2:**
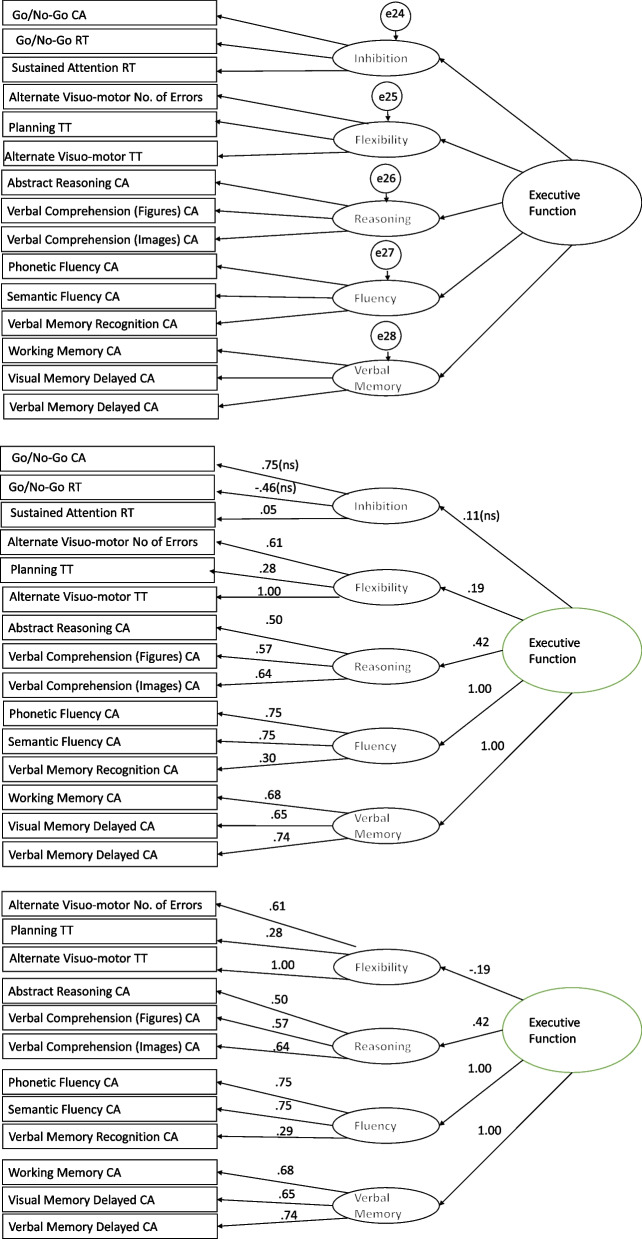
1: Five Factor Executive Function Model (χ2 (100, *N* = 604) = 245.55, *p* < 001, RMSEA = .049, CFI = .908, TLI = .875). 2 Five Factor Executive Function Model (χ2 (101, *N* = 604) = 205.73, *p* < .001, RMSEA = .041, CFI = .934, TLI = .911) ns – not significant. 3 Four Factor Executive Function Model (χ2 (51, *n* = 604) = 135.57, *p* < .001, RMSEA = .052, CFI = .944, TLI = .914)

A second-order model with Executive Functioning as a second-order latent factor and five first-order latent factors (i.e., Fluency, Reasoning, Memory, Inhibition and Flexibility) measured by the specific the BENCI subtests (Fig. 2) was specified and tested with the pooled sample including missingness handled by Full Information Maximum Likelihood. The model fit indexes suggested a good fitting model (χ2 (100, *N *= 604) = 245.55, *p* < 001, RMSEA = 0.049, CFI = 0.908, TLI = 0.875). However, this model had several issues. First, the Fluency factor was estimated to have a negative residual variance that we fixed at zero. Second, in this revised model, the Verbal memory factor also yielded an estimate negative residual variance that we treated similarly by fixing it at zero. Third, in the third model, the residual variance of the Alternate Visuo-motor total time also needed to be fixed to zero. Next, we considered modification indices and found that the model could be improved if we included a covariance between the residuals of Reasoning and Flexibility and between the residuals of Semantic Fluency correct answers and Verbal Memory Recognition correct answers. This further modified model showed an acceptable fit (χ^2^ (101, *N* = 604) = 205.73, *p* < 0.001, RMSEA = 0.041, CFI = 0.934, TLI = 0.911). Figure 2 presents the standardized factor loadings. An inspection of this model showed that not all indicators of Inhibition (Go No Go RT = λ -0.46; Go No Go CA = λ 0.74) had significant loadings on their respective factor, indicating that these specific tests did not measure Inhibition as intended (Fig. 2). It also showed that the latent factor of Inhibition did not load on the Executive Functioning factor. Therefore, we removed the Inhibition factor together with its indicators and tested a second-order factor with only four factors. This model fitted well (χ^2^ (51, *N* = 604) = 135.57, *p* < 0.001, RMSEA = 0.052, CFI = 0.944, TLI = 0.914). Figure 2 presents the factor loadings of this model. Therefore, the five components of Executive Functioning as validated before did not all show up in the Kenyan sample, while Executive functioning comprised of fluency, reasoning, verbal memory, and flexibility was found to fit well in the Kenyan sample. The final model with four factors each measuring executive functioning supports the construct validity for the BENCI battery, despite Heywood cases on the Alternative Visuo-motor subtest.

AMOS treats missing data using full information maximum likelihood, which is considered a robust method for treating missing data. However, we checked whether model fit would be affected when using a dataset with no missing data. On running the model with no missing data, the model fit was excellent (χ^2^ (51, *N* = 327) = 64.07, *p* > 0.05, RMSEA = 0.028, CFI = 0.968, TLI = 0.958). This shows that the BENCI does have good construct validity though some changes in some test items and instructions are needed in future revisions of some subtests.

#### Measurement invariance

**Fig. 3:**
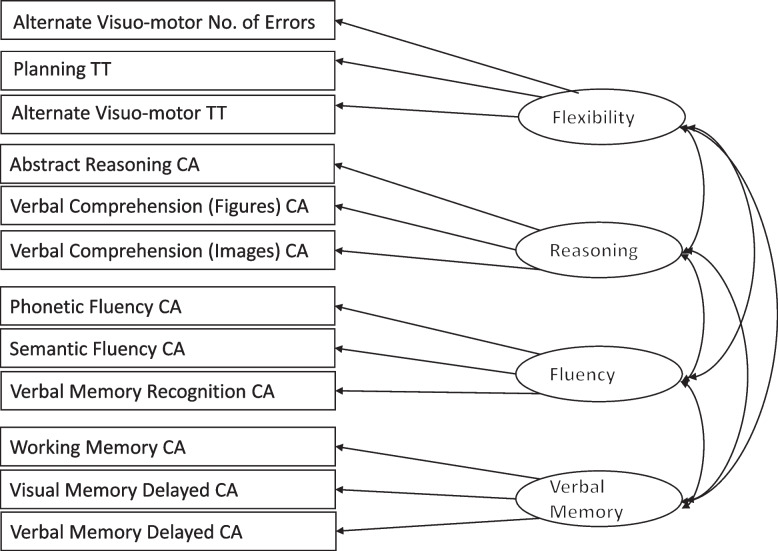
Four Factor First Order Model (χ2 (47, *n* = 604) = 107.76, *p* < 001, RMSEA = .046, CFI = .960, TLI = .933)

We set out to test whether the BENCI behaves the same way across the HIV-positive (*N* = 274) and HIV-negative groups (*N* = 330) using measurement invariance testing with multi-group confirmatory factor analysis. We used the factor model that was identified as having an excellent fit using the pooled sample as the basis and modified it to have only the four correlated first-order factors (i.e., Fluency, Reasoning, Memory, and Flexibility, each of them had their observed indicators) but no second-order factor (which is not required for testing measurement invariance). The model fit was excellent (χ^2^ (47, *n *= 604) = 107.76, *p* < 001, RMSEA = 0.046, CFI = 0.960, TLI = 0.933) as shown in Fig. 3.

We first tested for configural invariance where all factor loading, item intercepts and residual parameters were freely estimated. The model fit indexes suggested a well-fitting model (χ^2^ (94, *N* = 604) = 175.09, *p* < 0.001, RMSEA = 0.038, CFI = 0.941, TLI = 0.902). The factor loadings of all the indicators in both groups were significant.

We then specified a model for metric invariance where all the factor loadings were restrained to be the same across the two groups and all the other parameters were freely estimated. This model had a good fit (χ^2^ (102, *N* = 604) = 198.35, *p* < 0.001, RMSEA = 0.040, CFI = 0.930, TLI = 0.893). On comparing the configural to the metric invariance model, we found that there was no statistically significant difference between the chi-square values, suggesting that the metric invariance was supported (Δχ^2^ = 23.26, DF = 8, *p* = 0.003). This meant that the factor loadings were invariant and the indicator items across groups have the same associations with the latent constructs. Differences in other fit indexes also showed that the metric invariance was tenable (ΔCFI from configural to metric model < 0.01).

A scalar invariance model was then specified where the item intercepts and factor loadings were restrained to be the same across groups, while the latent mean of the latent factors in the HIV-positive group was released (with an aim to check latent mean differences in flexibility, fluency, verbal memory, and reasoning). This model had a poorer fit compared to the metric invariance model (χ^2^ (110, *N* = 604) = 245.12, *p* < 0.001, RMSEA = 0.045, CFI = 0.901, TLI = 0.860). On comparing this scalar invariance model to the metric invariance model, there was a worsening fit due to constraints on the intercepts; this was due to a statistically significant difference between the chi-square values of the scalar invariance and metric invariance model (Δχ^2^ = 46.77, DF = 8, *p* < 0.001). The CFI difference also showed that the scalar invariance was not holding across all subtests (ΔCFI = 0.029). This indicates that some intercepts were not invariant and that these subtests are uniformly biased.

Using modification indices, we then specified a partial scalar invariance model where we constrained one intercept for each indicator at a time and tested whether this restraint resulted in a significant chi-square difference. For items for Verbal Comprehension (figures) CA and Visual Memory Delayed CA, the tests showed significant chi-square difference hence we freely estimated these two intercepts across groups while holding the rest of the intercepts and factor loadings to be the same across groups. This partially invariant model fitted well (χ^2^ (108, *N* = 604) = 218.38, *p* < 0.001, RMSEA = 0.041, CFI = 0.920, TLI = 0.884). The fit for the partial scalar invariance was better than the strict scalar invariance, and the difference between the chi square values between this model and the metric invariance model shows that partial scalar invariance fits reasonably well (Δ χ^2^ = 20.03, DF = 6, *p* > 0.001). The CFI difference also showed that the partial scalar invariance was tenable (ΔCFI 0.010).

To summarize the series of measurement invariance tests, we conclude that metric invariance is achieved indicating that factor loadings of the BENCI are comparable across the HIV-positive and HIV-negative samples, and we can compare the association of the BENCI with other invariant constructs across the two groups, but not the mean comparisons of Verbal Comprehension (figures) CA and Visual Memory Delayed CA. These subtests are not well-calibrated. A partially scalar invariant model fitted the data reasonably well meaning you could compare mean difference for most of the subtests with caution for Verbal Comprehension Figures CA and Visual Memory Delayed CA.

## Discussion

This study aimed to validate the BENCI battery in Kenya with children living with HIV and those not living with HIV and contribute to a toolset of evaluation tests for primary school students in Kenya and other similar settings. There were four main analyses to address internal consistency, test–retest reliability, convergent validity, and construct validity among 6 to 14-year-olds. The adaptation of the English version of the BENCI resulted in a battery with good test–retest and validity checks. We discuss each finding and its implications in detail.

### Reliability

Some subtests were found to have floor effects due to having too many difficult items while others had ceiling effects due to having too many easy items. Too few and easy items resulted in ceiling effects for the language tests. The BENCI’s subtests showed poor to excellent internal consistency with most subtests showing higher alpha values for the HIV-positive group than the school sample. This was likely caused by smaller attenuation effects in the subtests with ceiling effects or the HIV-positive group showed more variation in true scores leading to higher Alphas as seen in the N-back working memory test [31]. The internal consistencies in our study were similar albeit slightly lower than those found in the Moroccan sample [20], possibly because the level of difficulty of the test items suited the younger cohort in the Moroccan sample better than in our data. This points to the need to develop age-appropriate norms and to add items with age-suitable difficulties in future revisions.

Our results for the BENCI test–retest reliability were fairly similar to a previous study conducted in Morocco [20]. The Arabic adaptation of the same tool reported Intraclass correlation to range from -0.23 to 0.81, similar to our study [32]. However, the poor test–retest reliability of the reasoning test could be due to the relatively long-time interval between the two assessments in our study as a longer interval may create changes in the construct [33]. It is possible that the respondents were thinking about the test items more often than before the first administration [33]. The latter is more likely with children who have high mental imagery skills meaning they are likely to think about the test items quite often and grow familiar with them and forthwith give different responses in the second assessment [34]. A child may respond substantially different in a language test whose retest is one year compared to verbal memory because their language ability has improved well past their memory ability. Studies on cognitive tests have had a re-test time interval of 15 to 60 days though there were recommendations for within a 14-day lapse of time especially for tests such as visual memory which would lose reliability over longer durations [20, 33, 35–37]. However, some studies have shown that for verbal memory and visual motor speed tests, the test–retest reliability with a one-year time-lapse remains stable while for language tests a recommendation for not less than 14 days has been made [36, 38]. The mixed results in our study suggest that the test domains and time lapses play a role here [37]. Our test–retest results in attention tests are also similar to those of other studies that show higher reliability in attention speed tests compared to accuracy tests [39]. Tests that call for speed over accuracy have been found to have high reliability than those that call for accuracy over speed [39].

### Convergent validity

BENCI attention tests do not correlate with Kilifi’s People Search and Forward Digit Span as expected, but they showed convergence with tests that had attention components. Studies have cited the tendency of attention tests to confound with other cognitive functions [40–42]. In our study, similar administration processes between tests with attentional components could have contributed to convergence as seen in the BENCI’s Working Memory test with Kilifi’s People Search test. These two are attentional control tasks as they call for a response to correct stimuli during incorrect stimulus thereby inhibiting a response. Correlations between attention tests have been found to support convergent validity with a range from low- to-moderate. Speed measures have higher significant correlations compared to accuracy attention measures [39, 43]. In our study, however, the BENCI attention accuracy tests showed moderate convergent validity while attention speed measures showed weak convergent validity. Poor convergent validity between some attention tests has been documented in other studies [40]. In the memory domain, BENCI’s working memory and Kilifi’s people search correlated well, a finding that has also been found in other studies comparing working memory tests to attention tests [37, 41, 42].

BENCI’s Visual Memory test showed a weak correlation with Kilifi Toolkit’s Nonverbal Selective Reminding Memory Test (NVRST). The administration is similar between these two tests. An explanation for this could be found in studies showing the impact of familiarity with the tools on scoring. In our study, the NVRST test involved memorizing the shape formed by a set of 8 dots and then replicating the shape by placing a marble on a set of dots. In the BENCI version, the child was supposed to memorize several images and then correctly point them out when shown amidst a set of other pictures; a task that would involve other cognitive functions such as visual-motor coordination. Pointing out pictures is a familiar learning concept in the Kenyan context. This is because among the methods used in teaching preschoolers is by pointing out images and encouraging the children to read and memorize their names. The administration was fairly similar but their scores in terms of correct answers were not highly correlated. Probably other psychological processes are involved in the BENCI subtest that are not in the Kilifi subtest. There are some studies that have found a similar lack of correlation between tests. In a study done in Zambia, a non-verbal test called draw-a-person was locally adapted and the two tests, the original and adapted one, were compared and found to not be correlated [44]. However, when the ratings were done by adults and correlated to educational outcomes, the two tests had significant correlations. Further research can explore similar comparisons between uncorrelated tests to find out if other psychological processes are involved. Such an evaluation could be similar to the one conducted in the Zambian study. This is in trying to find out whether the BENCI visual memory test expectations do truly reflect the cultural indicators for non-verbal memory. However, the NVRST in the form of Children’s Memory Scale (CMS) dot location subtest has also been found not to have significant correlations with the Lebby-Asbell Neurocognitive Screening Examination (LANSE) visual memory test [42]. In addition, NVRST administration involves visuomotor coordination and other cognitive functions in addition to memory.

Computerized assessments are preferred due to ease of administration and scoring as well as precision [37]. However, Kenyan children are not very used to computerized assessments and a lack of familiarity may introduce variance in test scores that are not related to the construct being measured. Some of the factors that have been known to introduce construct irrelevant variance with computerized assessments include proficiency with the computer-based tests, ease of interaction with the platform, speediness of the tests and test-taker’s anxiety [45]. Some administration processes, such as tasks calling for inhibitory control, within the tablet may affect some domains more than others [46]. The lack of familiarity and some administration processes associated with tablet-based testing could affect convergence validity when compared to some paper-based tests. However, there are some studies that have shown no significant differences in test performance between tests using computer-based platforms and those using paper-based ones meaning that variation in convergence may apply to some tests more than others [46]. To reduce variation in some of these tests, studies have suggested several approaches including reducing the difficulty level of computer-based tests as well as clarifying the relationship between tasks and the expected test takers performance [45, 47]. It is however, beyond the objectives of this study to investigate approaches that would have worked best in reducing validity variance between the BENCI and Kilifi toolkit. These are next level questions to consider.

The lack of convergence in some tests may also be contributed to by lack of a common construct between some of the BENCI and Kilifi toolkit tests. Since the latter is the gold standard, comparing it to a test that that does not capture the same constructs may give us erroneous findings. Differences in correlations between measures have been found to increase when comparisons are made to alternate measures with low convergence validity [47]. Improvements and adaptations of some of the BENCI tests may improve convergence with the Kilifi toolkit tests.

### The BENCI functionality in age and HIV groups

The BENCI highlighted clear mean differences between the HIV-positive and HIV-negative groups. Just as indicated in the BENCI results, tests can have mean differences but the score differences between the groups may not be significantly different as seen in the scores for correct answers in the inhibition test and time taken in the planning test. An earlier study showed that certain tests like inhibition and planning can have the ability to differentiate healthy from unhealthy populations but the difference in scoring within the tests may not be significantly different [48]. However, the BENCI did affirm what other studies have found that children living with disease score lower than children living without disease in tests of working memory, inhibition, memory, and planning among other cognitive functions [42, 48–50]. Moreover, taking more time when doing a test has been associated with taking more mental effort to achieve a desired outcome, in this case a correct response, entails a healthy approach to inhibitory tasks [30]. Better performance in correct answers is denoted by higher scores while in reaction time it is denoted by lower scores. Therefore, for children having high reaction time, performance will be regarded as poor. Overall, this is true when the dependent variable is time but not when it is accuracy. For example, higher reaction time is worse than a lower one in Selective Attention, Sustained Attention and Go/No-Go tests. These findings add to the body of literature on the significance of testing for cognitive deficiencies among unhealthy children.

### Construct validity

The planning test did not have significant loading on the inhibition factor in the pooled sample and subsequently, this factor did not load well onto executive function. This has not been the case in another Sub-Saharan African study that supported the construct validity of a planning test [51]. Inhibitory control has been found to be higher in children within settings that emphasize obedience and self-control such as East Asian countries and been found to be lower, to a point where there are no significant age differences, among children in developing countries and communities [52]. The study also reported cross-national differences in inhibition, shifting, and updating. We would then expect the children in this study to have the BENCI inhibition tests to load onto executive function just like other western adapted tests have done in a sub-Saharan setting. This is more so since inhibition tends to develop rapidly among younger children hence, we would not expect a lack of this cognitive function among 6- to 14-year-olds even though inhibitory control tends to mature at adolescence [30, 53]. However, studies looking into whether maturity of inhibitory control affects how well the function can load into an executive function model may clarify the results we found in this study. Observations of the school and home executive function stimulation activities give a broader picture of the activities emphasized and how they encourage inhibitory control development. These observations could be integrated in further research with the BENCI. Flexibility on the other hand builds developmentally onto inhibition and loaded well on executive function. This finding does not reflect the arguments pointed out earlier on inhibition. Inhibition is a first-order component that appears around 6–8 years and flexibility is a second-order component that appears later in development [54]. Since flexibility loaded well onto executive function, the lack of significant loadings in the inhibition construct could potentially be because of the lack of culturally aligned items in the inhibition tests or a problem with instructions. The findings in the construct validity indicators call for a developmental approach when interpreting scores and the need to norm the BENCI for age groups.

The BENCI also showed support for metric and partial scalar invariance as opposed to strict scalar invariance. This means that the BENCI items are loaded onto the latent factors similarly across groups, hence can be compared across the groups. The same applies to items per subtest. However, comparability of means between the latent factors was not supported in its entirety meaning that we cannot compare the means of fluency, flexibility, verbal memory, and reasoning across the groups. We can choose to create separate norms for HIV + and HIV- groups since the tests behave differently in the two groups, but this will not give us an opportunity to compare performance. One of the options that can enable performance comparison is to create norms with the healthy and optimally functioning group but caution should be integrated when norming for Verbal Comprehension Figures and Visual Memory Delayed tests. We may underestimate or overestimate between-group abilities due to miscalibration of the tests and the results may be marred with measurement bias. This means that we may not have true between-groups construct differences due to other construct irrelevant variables causing differences in test scores. In this case, we may choose to correct for intercept differences during norming by estimating their effect sizes and relating this to effects on the norm scores [55]. As an alternative, we can choose to carry out a study on why the two tests are biased and correct for any item level (attenuation effects in Verbal Comprehension Figure). We are yet to come across a study that investigates measurement invariance of a neurocognitive tool in Kenya and its regions. Children studies that we have come across are based in high income countries [56] and cannot be compared to our setting due to different group dynamics and cultural dynamics that underlie cognitive performance and developed test items [24].

### Limitations

In this study, one drawback was that the results could only be generalized in a community setting and not a clinical one. We could not find comparison tests for some domains due to the limited availability of validated tools within the Kenyan culture.

The study also noted that some subtests had floor and ceiling effects, which compromised the interpretation of other findings. In this case, any results pertaining to the subtests having ceiling and floor effects should be interpreted with caution. Moreover, further studies may revise the tests by perhaps adding more items to the tests with ceiling effects and decreasing the difficulty of the items in the tests with floor effects so as to match the difficulty to ability level and reduce attenuation effects. In addition, age-appropriate norms for the subtests should be considered.

The methods used to capture reaction time and total time may not have been completely accurate because the paper–pencil tests used a stopwatch that is prone to administration errors while the IPad-based tests used an internally configured watch. In the paper-based tests, errors may be integrated when timing is not stopped immediately a task is completed or when an administrator gives more time for task completion than would be required. These can create systematic or random measurement errors where the latter could suppress correlations. This may have been the case in convergent validity where random measurement could have suppressed some correlations. Nevertheless, the possibility of errors in paper-based tools is another reason to prefer automated computerized tests with internalized and consistent timing across participants.

## Conclusion

The Spanish version of the BENCI was successfully adapted to English, and its psychometric checks showed that it had good convergent validity in reasoning and some memory and inhibition tests. However, further research is needed to fully understand the non-verbal memory, working memory and flexibility tests from a convergent validity view. The BENCI was also found to have good discriminant validity with only a few tests not showing a significant difference between the case and control populations. Construct validity showed good goodness of fit indicators though the inhibition did not load onto executive function as expected. Future language adaptations can consider Kiswahili translations which is Kenya’s national language.

HIV is a known risk factor for poor neurocognitive outcomes due to its negative impact of CNS and exposure to a host of negative psychosocial factors. We therefore hypothesized that children living with HIV would perform worse than those who are uninfected. Confirming our hypothesis, children living with HIV performed significantly worse than those who were uninfected, thus showing that the BENCI is sensitive to a well-documented biological risk factor.

## Supplementary Information

**Additional file 1: Supplementary Table 1.** Pilot Study BENCI Observations, Respondents Feedback and Researchers Recommendations.

## Data Availability

All the data generated in the study as well as materials will be made openly available through DataverseNL. This is a GDPR compliant and publicly available repository that complies with FAIR (Findable, Accessible, Interoperable, Reusable) principles. A request through the corresponding author will have one access the data.

## Abbreviations

BENCI: Computerized Battery for Neuropsychological Evaluation of Children
HIV: Human Immuno-Deficiency Virus
ART: Antiretroviral Therapy
SSA: Sub-Saharan Africa
SPSS: Statistical Package for Social Sciences
KABC II: Kaufman Assessment Battery for Children Second Edition
DF: Degrees of Freedom
SQRT: Square Root
RMSEA: Root Mean Square Error of Approximation
CFI: Comparative Fit Index
TLI: Tucker-Lewis Index
ICC: Intraclass Correlation
CA: Correct Answers
RT: Reaction Time
TT: Time Taken
NACOSTI: The National Commission for Science Technology and Innovation
PAM-D: Partnerships for Mental Health Development in Sub-Saharan Africa
NIMH: National Institute of Mental Health
GDPR: General Data Protection Regulation
NVRST: Nonverbal Selective Reminding Memory Test
CMS: Children’s Memory Scale
LANSE: Lebby-Asbell Neurocognitive Screening Examination
CNT: Contigency Naming Test
VLL: Verbal List Learning
SOPT: Self-ordered Pointing Test
RPM: Raven’s Progressive Matrix
ANT: Attentional Network Task
Pediatric ImPACT: Pediatric Immediate Post Concussion Assessment and Cognitive Testing

## Footnotes

1 The original plan was to include children aged 8 – 11 years but we included also 6, 7 and 12 – 14 year olds (*N* = 52, 9.5%) because they were in the same grade.
