# Supplementary material for: Psychometric evaluation of the computerized battery for neuropsychological evaluation of children (BENCI) among school aged children in the context of HIV in an urban Kenyan setting
Source: BMC Psychiatry. 2023 May 29;23:373. doi: 10.1186/s12888-023-04880-z (PMC10226211; doi:10.1186/s12888-023-04880-z)
Supplement: Supplementary file 1 — Additional file 1: Supplementary Table 1. Pilot Study BENCI Observations, Respondents Feedback and Researchers Recommendations. [file 12888_2023_4880_MOESM1_ESM.docx]

Supplementary Table 1: Pilot Study BENCI Observations, Respondents Feedback and Researchers Recommendations

| **Subtest** | **Observations by Two Testers** | **Recommendations by the Research Team** |
| --- | --- | --- |
| Verbal Comprehension - Figures | a. The instructions do not prepare some children well for the test. The instructions orient the child to tapping either to the right or left of an image. However, the test requires the child to tap a figure with certain traits. Probably the wording should be changed to exude this.  b. Some respondents tend to tap on 2-3 figures rapidly before the next instructions. The instructions should therefore indicate that only one figure should be tapped on per instruction.  c. I didn’t have a problem on my end in regards to the instructions. I think the instructions are simple enough to follow once they see the task itself. | 1. Maybe in the instructions instead of saying ‘figures’ we use the word ‘shapes’ or ‘images’ cause that is what we are taught they are called in school. 2. Provide new instructions adequate for Kenyan children “Follow the instructions indicated below. If the instructions ask you to tap at the right or the left of the images, you will have to tap at your right or your left. Press the START button to launch the test” 3. We can use the training section of the test to succinctly orient the children to the test. |
| Working Memory | 1. Clear instructions and intense training needed as younger children tend to apply the same instructions as the ones in previous tests precluding the grouping bit. Also inform the child that she/he needs to wait for the microphone. 2. I feel that part of the instructions that was difficult for them to understand was that they are meant to repeat either colors first only and then the numbers next cause they just repeated what they heard. | 1. I think that we have to fix the order, for instance, first number and then colors. I think that this is important for the standardization of the test. 2. Make the instructions clear and use the training option to evaluate whether the child has understood the instructions. |
| Verbal Memory with Delayed Trial | 1. Have the children keep their hands off the screen unless they are responding. Double tapping the screen or idly passing their fingers on the screen may interfere with preferred responses. 2. The instructions in between are not in English. 3. The instructions of ‘Yes/No’ is not in English | 1. Advice the children to keep their hands off the screen if not responding. 2. Translation “Next, you will hear a list of words. You have to tap "yes" if that word was in the list of word that you listen before or tap "no" if not.” |
| Visual Memory | 1. Screen is very sensitive to any tapping so child’s hands should be off the screen if not responding. 2. Some of the images that respondents had difficulty naming or telling apart include Cloud/bush and hair/head 3. There are instructions still in Spanish. 4. I didn’t experience a challenge with this task. 5. The instructions of ‘Yes/No’ is not in English. | 1. Advice the children to keep their hands off the screen if not responding. 2. Images with two possible names, for example hair/head. In this case, we only have to specify in the manual that hair or head are correct answers for Kenyan children. 3. Translation “Next, you will see several images. You have to tap "yes" if that image was in the list of images that you saw before or tap "no" if not.” |
| Planning | 1. Two children found it really hard to reason out what was needed. I will explore further to find out an easier way of doing this. Probably simpler English would do. 2. When reading out the instructions to them (in kiswahili), I used examples that they knew. Instead of ‘theme park’, I talked of ‘Uhuru Park’ which most are familiar with, and instead of ‘tokens’ I use the word ‘shillings’. | 1. ‘Shillings’ is more comprehensible than ‘Tokens’. Also call it a ‘play park’. |
| Continuous Performance | 1. Two children found the test too long and had to be encouraged to finish. One gave up and did other tests after which she was encouraged to finish. 2. I timed this test and it took 9 minutes. The test is configured to have 3 blocks. | 1. I realize that is a very boring test but it should to be so. The main objective is to sustain attention in a low demanding task. Children with attention problems can sustain attention during the first minutes but after that, they fail the task and produce more errors. However, if you feel that it is too long, we could reduce the duration. In my opinion, it should be 8-10 minutes at least. Nine minutes is quite acceptable even for 6 years old. 2. If reducing the duration will compromise the test then we can maintain the configuration and schedule to have a break right before this test. |
| Alternate Visual Motor | 1. The instructions indicate that there will be numbers and letters but the test has numbers only with some of the numbers inside squares and others inside circles. The previous test before upgrading to the app version had test as per instructions (letters and numbers). 2. Even in Kiswahili these instructions are unclear, but the training helped a little bit. | 1. Use the settings to reconfigure this to animals and fruits instead. Children can start with the animals first then the fruits. 2. Let’s do more training. |
| Abstract reasoning | 1. Translation needs to be done ‘*completa la serie’*. | 1. Translation “Complete the pattern” |
| Go-No-Go | 1. I think the instructions are simple enough for one's understanding. However, the sound that comes as a result of tapping the elements confuses the children such that when the sound they are supposed to be watching out for comes, they are unable to hear it. I understand, however, that it is part of the test. A lot of training needs to be done so that the child gets to familiarize themselves with the sound. 2. I gave all my instructions to the children in Kiswahili because they were all from poverty area and I realized that the English instructions would not be understood easily especially those tasks with a lot of wording. E.g. go-no-go, working memory, visual and verbal memory with delayed trial, planning. | 1. Even as we are translating the instructions, we need to make them simple enough for them to understand, and ensure that the task is also understood. |
| Semantic & Phonetic Fluency | 1. The training in phonetic fluency and semantic fluency uses examples that are also used in the test. Is this appropriate? | 1. This is to be fixed. The training set should be different from the testing set. |
